# Hypothalamic *KiSS1/GPR54* Gene Expressions and Luteinizing Hormone Plasma Secretion in Morphine Treated Male Rats

**DOI:** 10.22074/ijfs.2018.5332

**Published:** 2018-06-20

**Authors:** Homayoun Khazali, Fariba Mahmoudi, Mahyar Janahmadi

**Affiliations:** 1 Faculty of Biological Sciences, Shahid Beheshti University, Tehran, Iran; 2 Faculty of Sciences, University of Mohaghegh Ardabili, Ardabil, Iran; 3 Neurophysiology Reseaech Center and Department of Physiology, Medical School, Shahid Beheshti University of Medical Science, Tehran, Iran

**Keywords:** *GPR54*, *KiSS1*, Luteinizing Hormone, Morphine

## Abstract

**Background:**

The inhibitory effects of morphine and the stimulatory influence of kisspeptin signaling have been demonstrated on gonadotropin releasing hormone (GnRH)/luteinizing hormone (LH) release. Hypothalamic kisspeptin is involved in relaying the environmental and metabolic information to reproductive axis. In the present study, the role of kisspeptin/ *GPR54* signaling system was investigated on relaying the inhibitory influences of morphine on LH hormone secretion.

**Materials and Methods:**

In this experimental study, 55 wistar male rats weighing 230-250 g were sub-grouped in 11 groups (in each group n=5) receiving saline, kisspeptin (1 nmol), peptide234 (P234, 1 nmol), morphine (5 mg/kg), naloxone (2 mg/kg), kisspeptin/P234, morphine/naloxone, kisspeptin/morphine, kisspeptin/naloxone, P234/morphine or P234/naloxone respectively. Blood samples were collected via tail vein. Mean plasma (LH) concentrations and mean relative *KiSS1* or *GPR54* mRNA levels were determined by radioimmunoassay (RIA) and real time reverse transcriptase-polymerase chain reaction (RT-PCR), respectivwely.

**Results:**

Morphine significantly decreased mean plasma LH concentration and mean relative *KiSS1* gene expression compared to saline; while it did not significantly decrease mean relative *GPR54* gene expression compared to saline. Naloxone significant increased mean LH level and mean relative *KiSS1* gene expression compared to saline; while it did not significantly increase mean relative *GPR54* gene expression compared to saline. Injections of kisspeptin plus morphine significantly increased mean LH concentration compared to saline or morphine, while simultaneous infusions of them significantly declined mean plasma LH level compared to kisspeptin. In kisspeptin/naloxone group mean plasma LH level was significantly increased compared to saline or naloxone. Co-administration of P234/morphine significantly decreased mean LH concentration compared to saline.

**Conclusion:**

Down regulation of *KiSS1* gene expression may be partly involved in the mediating the inhibitory effects of morphine on reproductive axis.

## Introduction

kisspeptin/*GPR54* signaling pathway has a therapeutic potential, as regulator of gonadotropin releasing hormone (GnRH)/luteinizing hormone (LH) release and gonadal steroid hormone secretions. G protein-coupled receptor, *GPR54*, is expressed in GnRH neurons and normal pubertal development, while sexual function is also dependent to normal actions of it (1, 2). Reproductive process is disrupted by the mutations of *GPR54* receptor or kisspeptin genes (3). Kisspeptin analogues are introduced as endogenous ligand for *GPR54* receptor and four types of kisspeptin (kisspeptins 10, 13, 14 and 54) have similar affinity to this receptor. They induce puberty and peripheral or central injections of them increase the GnRH/LH release and plasma gonadal steroid (1-4). Infusions of peptide 234 (P234) also block the stimulatory effects of kisspeptin on LH secretion (5).

Opioids suppress the reproductive process, resulting in hypogonadotropic hypogonadism (HH) dominantly via inhibiting the hypothalamus-pituitary-gonadal (HPG) axis rather than direct effects on pituitary or testes (6). Morphine, as an alkaloid extracted from poppy plant, is extremely used as drug abuse and drugs for the suppressing pain. Injections of morphine decrease the secretion of GnRH and LH via binding to opioid µ-type recep. tors (6-8). However, Aloisi and her colleague reported that morphine treatment may play a role in declining the mean plasma testosterone level by increasing peripheral testosterone metabolism in testes, liver and hypothalamus (9). It has also been found that naloxone, acting as the antagonist of µ-opioid receptor, blocks the influences of morphine on the HPG axis. In contrast, it induces puberty and improves the GnRH/LH as well as gonadal hormone secretions in males and females of different species (10).

Opioids receptors are not directly expressed on GnRH neurons and they exert their inhibitory influences on the reproductive axis via different interneurons pathways (11). In addition, several studies have established that kisspeptin has a crucial role in relaying the central or peripheral information to the reproductive axis (12-16). In order to the significant importance of physiological action of kisspeptin/ *GPR54* signaling pathway for controlling GnRH/LH release and considering the clinical overuse of opioid drugs, the present study aimed to investigate that if the level of kisspeptin/ *GPR54* signaling system activity may be partly involved in the morphine- induced decline of LH mean plasma levels.

## Materials and Methods

In this experimental research, three months old male wistar rats (n=55), weighing 230-250 g (provided by the Center of Neuroscience Research of Shahid Beheshti University, Tehran, Iran), were housed in the cages under controlled temperature (22 ± 2°C) and light (12 hours light/dark cycle). Animals had always free access to food and water. All procedures for the maintenance and use of experimental animals were executed with the Guide for the Care and Use of Laboratory Animals (National Institute of Health Publication No. 80-23, revised 1996, Iran) and were approved by the Ethical Committee of Neuroscience Research Center of Shahid Beheshti University of Medical Sciences (Tehran, Iran).

### Intra cerebral ventricular cannulation and injections

Animals were anesthetized by intraperitoneal (IP) injections of a mixture of ketamine and xylezine (ketamine 80 mg/kg bodyweight+xylezine 10 mg/kg bodyweight), a 22-gauge stainless cannulae was implanted in the third cerebral ventricle according to coordinates of Paxinos and Watson Atlas [anterior posterior (AP)=-2.3, midline (ML)=0.0, dorsoventral (DV)=6.5] (17). After one week, 55 rats were divided into 11 groups (5 in each group), receiving drugs as mentioned in the Table 1.

**Table 1:** Received drugs (name and dose) in each groups (n=5)


Groups	

1	Saline (3 µl, ICV)/saline (200 µl, SC)
2	Kisspeptin (1 nmol/3 µl, ICV)/saline (200 µl, SC)
3	P234 (1 nmol/3 µl, ICV)/saline (200 µl, SC)
4	Kisspeptin (1 nmol/1.5 µl, ICV)+P234 (1 nmol/1.5 µl, ICV)/saline (200 µl, SC)
5	Saline (3 µl, ICV)/morphine (5 mg/kg, 200 µl, SC)
6	Saline (3 µl, ICV)/naloxone (2 mg/kg, 200 µl, SC)
7	Saline (3 µl, ICV)/naloxone(2 mg/kg, 100µl, SC)+morphine (5 mg/kg, 100 µl, SC)
8	Kisspeotin (1 nmol/3 µl, ICV)/morphine (5 mg/kg, 200 µl, SC)
9	Kisspeptin (1 nmol/3 µl, ICV)/naloxone (2 mg/kg, 200 µl, SC)
10	P234 (1 nmol/3 µl, ICV)/morphine (5 mg/kg, 200 µl, SC)
11	P234 (1 nmol/3 µl, ICV)/morphine (5 mg/kg, 200 µl, SC)


ICV; Intra cerebral ventricular and SC; Subcutaneously.

Kisspeptin10 (Ana Spec Co., USA) and P234 (Phoenix Pharmaceuticals Inc., USA) were dissolved in distilled water and injected intra third cerebral ventricle by using Hamilton micro syringe at 09:00- 9:30. Morphine sulfate (Temad Co., Iran) and naloxone hydrochloride (Toliddaru Co., Iran) were dissolved in distilled water and injected SC by an insulin syringe at 09:00-9:30. In simultaneous groups, naloxone was injected 15 minutes before morphine injections. The time of blood sampling as well as kisspeptin, naloxone or morphine doses was chosen based on our laboratory and other previous studies reporting the stimulatory or inhibitory effects of these drugs on the reproductive axis, respectively (2, 3, 9, 10).

### Hormone assays

Blood samples were collected in a volume of 0.5 cc at 60 minutes following the injections via tail vein. Heparin was added to the samples to prevent clotting. Blood samples were immediately centrifuged for 15 minutes at 3000 rpm and the plasma samples were stored at -20°C until assayed for LH concentration. Mean plasma LH concentration was measured by using rat LH kit and the method of the radioimmunoassay (RIA, Institute of Isotopes Co, LT'D, Hungary). Sensitivity and intraassay of the kit were 0.09 ng/ml and 4.61%, respectively. 

### Microdissections and total RNA extraction 

Four hours after injections, the rats were sacrificed by decapitation and the brains were immediately autopsied. The brains were placed ventral side up, anterior coronal slices were cut from 1 mm anterior to optic chiasm. The slices were then dissected laterally up to the hypothalamic sulci and posterior coronal slices were cut posterior to the mammillary bodies (17). The samples were frozen by liquid nitrogen and stored at -80°C for determination of mRNA levels. Total RNA was isolated from individual frozen samples using the acid guanidinium thiocyanate- phenol-chloroform extraction method, according to PureZol manufacturer instruction (Bio RAD, USA). The quantification of each RNA sample was performed by measuring absorbance at 260 nm. The *GAPDH* gene was used to normalize the values obtained for each sample. 

### RNA analysis by real-time reverse transcriptase polymerase chain reaction 

Changes in the gene expression levels were determined by using the Corbett Real-Time PCR detection system Rotorgene 6000 (Qiagen Ltd, Germany). Total RNA (100 ng) was treated by DNaseI to remove residual genomic DNA according to manufacturer instruction (Thermo Scientific Inc., USA). Then, total RNA was further amplified in triplicate by using SYBR green I as fluorescent dye and one step quantitative reverse transcriptase RT-qPCR Master Mix Plus for SYBR Green I kit in a final volume of 25 µl according to manufacturer instruction (Eurogentec CO, USA). The PCR cycling conditions were as follows: reverse transcriptase step 48ºC for 30 minutes, 95ºC for 10 minutes, followed by 40 cycles of denaturation at 95ºC for 15 seconds, annealing at 54ºC (*KiSS1*), 54ºC (*GPR54*) and 58ºC (*GAPDH*) for 15 seconds and extension at 72ºC for 40 seconds. Specific oligo nucleotide sequences for sense and antisense primers were used as following:

*KiSS1*- F: 5'-AGCTGCTGCTTCTCCTCTGT-3'
  R: 5'-AGGCTTGCTCTCTGCATACC-3' (18)
*GPR54*- F: 5'-GGTGCTGGGAGACTTCATGT-3'
  R: 5'-AGTGGCACATGTGGCTTG-3' (18)
*GAPDH*- F: 5'-AAGAAGGTGGTGAAGCAGGCATC-3'
  R: 5'-CGAAGGTGGAAGAGTGGGAGTTG-3' (19).

*KiSS1*, *GPR54* and *GAPDH* amplified product lengths were 151, 72 and 112 base pairs, respectively. To ensure the specification of RT-qPCR products the melting curve for fragments were generated by the Rotorgene 6000 program and the PCR products were evaluated in 1.5% agarose gel electrophoresis. Calculation of the relative expression levels of targeted cDNAs were conducted based on the cycle threshold (C_t_) method. The C_t_ for each sample was calculated using the Corbett Real-Time PCR detection system software with an automatic fluorescence threshold (Rn) setting. Accordingly, fold expression of target mRNAs over the reference values was calculated by the equation 2^-ΔΔCt^.

### Statistical analysis

The results are presented as mean ± SEM. The data were analyzed by using SPSS software (version 16) and the one- way ANOVA followed by post hoc Tukey test. In all cases, statistical significance was defined by P<0.05.

## Results

Kisspeptin increased significantly the mean plasma LH concentration by 1.71 times compared to saline. P234 decreased mean plasma LH concentration by 0.12 compared to saline; however this decrease was not statistically significant. Simultaneous injection of kisspeptin and P234 increased the mean plasma LH concentration by 0.29 times compared to saline, while this increase was not statistically significant. In addition, injection of P234 solely or simultaneous injection of kisspeptin and P234 decreased significantly mean plasma LH concentration respectively by 0.67 or 0.52 times compared to kisspeptin. 

Morphine decreased significantly mean plasma LH concentration by 0.48 times compared to saline. Mean plasma LH concentration increased significantly following naloxone injection by 0.48 times compared to saline. Simultaneous injection of naloxone and morphine increased mean plasma LH concentration by 0.17 or 1.24 times compared to saline or morphine, respectively. This increase was not statistically significant compared to saline, while it was statistically significant compared to morphine. 

Co-administration of kisspeptin/morphine increased significantly mean plasma LH concentration by 0.73 or 2.32 times compared to saline or morphine, respectively. 

Additionally, co-administration of kisspeptin/morphine decreased significantly mean plasma LH concentration by 0.37 times compared to kisspeptin. Co-administration of kisspeptin/naloxone increased significantly mean plasma LH concentration by 2.12 or 5.04 times compared to saline or naloxone, respectively. 

**Fig.1:**
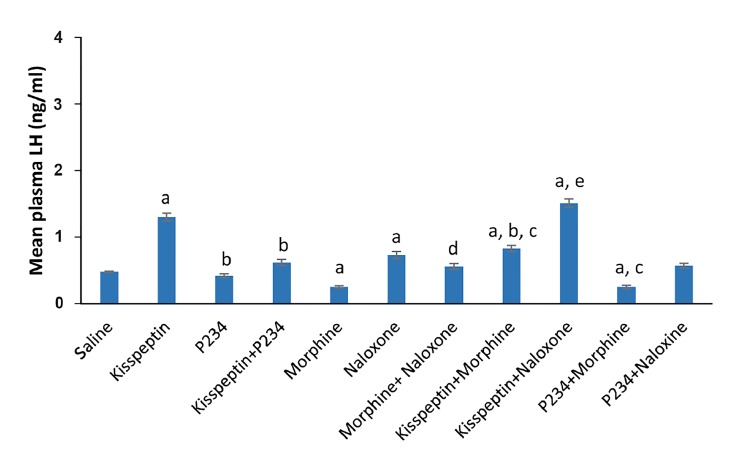
Effects of kisspeptin (1 nmol), P234 (1 nmol), 5 mg/kg morphine (MOR), 2 mg/kg naloxone (NAL) or co-administration of kisspeptin/morphine, kisspeptin/naloxone, P234/morphine or P234/naloxone on mean plasma LH concentration, in comparison with a; Saline, b; Kisspeptin, c; P234, d; Morphine, and e; Naloxone. Data are presented as mean ± SEM, P<0.05 and n=5 in each group.

Moreover, LH concentration was increased in kisspeptin/ naloxone group by 0.16 times compared to kisspeptin group, although this increase was not statistically significant. Co-administration of P234/morphine decreased mean plasma LH concentration by 0.5, 0.1 or 0.4 times compared to saline, morphine or P234, respectively. This decrease was statistically significant compared to saline or P234 (P<0.05, Fig .1), while it was not statistically significant in comparison with morphine. Co-administration of P234/naloxone increased mean plasma LH concentration by 0.18 times compared to saline, but this increase was not statistically significant. Furthermore, co-administration of P234/naloxone decreased mean plasma LH concentration by 0.21 times compared to naloxone, while this decrease was not statistically significant (Fig .1).

In addition, results showed that morphine induced a significant decrease in *KiSS1* mRNA expression levels in the hypothalamic samples compared to saline, naloxone or morphine plus naloxone injected groups. So that morphine decreased significantly mean relative *KiSS1* gene expression by 0.89, 0.93 or 0.85 times compared to saline, naloxone or morphine plus naloxone, respectively. Naloxone increased significantly mean relative *KiSS1* gene expression by 0.68, 14.27 or 1.21 times compared to saline, morphine or morphine+naloxone respectively.

**Fig.2:**
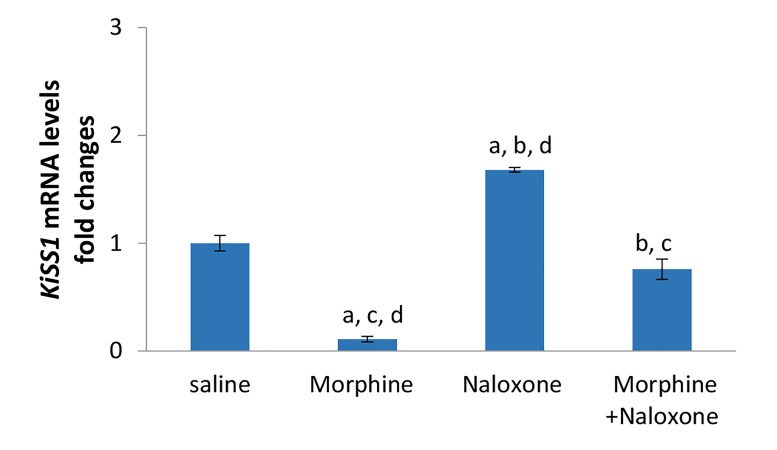
Effects of morphine (5 mg/kg), naloxone (2 mg/kg) or simultaneous injections of morphine and naloxone (n=5 in each group) on *KiSS1* mRNA expression in the hypothalamus of male rats. The cDNA amplified from *GAPDH* mRNA was used to normalize corresponding *KiSS1* results. The results are presented as mean ± SEM. In all cases P<0.05 was considered to be statistically significant. a; Compared to saline, b; Compared to morphine, c; Compared to naloxone, and d; Compared to morphine+naloxone.

**Fig.3:**
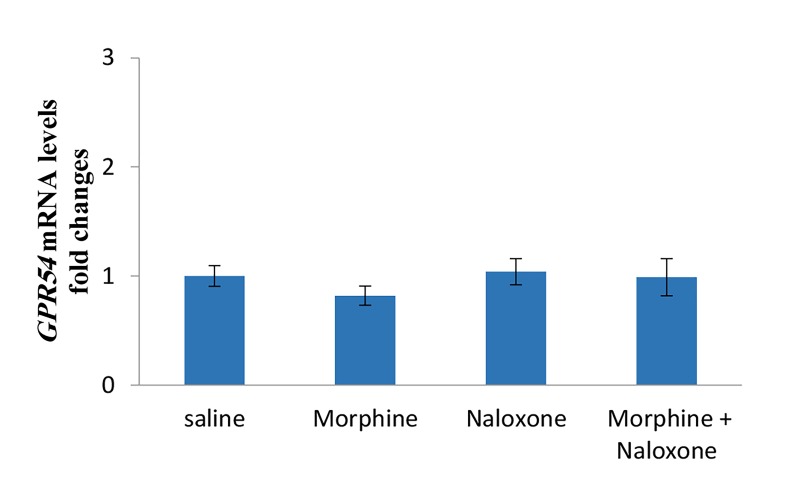
Effects of morphine (5 mg/kg), naloxone (2 mg/kg) or simultaneous injections of morphine and naloxone (n=5 in each group) on *GPR54* mRNA expression in the hypothalamus of male rats. The cDNA amplified from *GAPDH* mRNA was used to normalize corresponding *GPR54* results. The results are presented as mean ± SEM. In all cases P<0.05 was considered to be statistically significant.

In animals receiving naloxone+morphine, the mean relative *KiSS1* gene expression was decreased by 0.24 or 0.54 times compared to saline or naloxone, respectively. This decrease was not statistically significant compared to saline, while it was statistically significant compared to naloxone. Additionally, injections of naloxone+morphine increased significantly the mean relative *KiSS1* gene expression by 5.9 times compared to morphine (P<0.05, Fig .2). The mean relative *GPR54* gene expressions were not significantly influenced by the injections of morphine, naloxone or morphine+naloxone compared to saline group. Moreover, a significant decrease or increase was not observed on the *GPR54* mRNA levels between different groups (Fig .3).

## Discussion

The results showed that subcutaneous injection of naloxone or central injection of kisspeptin increased significantly the mean plasma LH concentration compared to saline, while subcutaneous injection of morphine significantly decreased it, in comparison with saline. These results are consistent with the other researches which established the stimulatory effects of naloxone (10), kisspeptin (1-5) or inhibitory effects of morphine on the sexual hormone secretions (6-9) and introduced them as important key regulators for controlling the HPG axis in the male and females of different species. 

In our previous studies, we showed that interaction of morphine/kisspeptin play a role in the regulating of mean plasma testosterone concentration in male rats (8). In this work, our results indicated that morphine injection attenuates the stimulatory effects of kisspeptin on mean plasma LH concentrations anf injection of kisspeptin+naloxone exerts an additive stimulatory effect on mean levels of LH, compared to naloxone. The precise molecular and central mechanisms underlying the effects of opioids on the reproduction neuroendocrine axis is not clear yet. 

However previous researches demonstrated that endogenous opioids, exogenous opiates (e.g. morphine) or their receptor antagonists influence the release of LH and subsequently gondal steroid hormones via indirect regulation of the hypothalamic GnRH release (11). However Kappa opioid receptors have been found on hypothalamic kisspeptin neurons of arcuate nucleus (ARC) (20), but mu opioid receptors mediating the physiological effects of ß-endorphin or morphine (21) are widely expressed in the brain stem and thalamic nuclei and lower levels expression of them has been reported in hypothalamus or GnRH neurons. Different signaling pathways supposed to be involved in mediating opioids indirect effects on the hypothalamic GnRH-producing neurons, which we could point to noradrenergic, dopaminergic or GABAergic neurons (11).

It is well established that more than 80% GnRH neurons express *GPR54* receptor and hypothalamic *KiSS1* has been proposed as key molecular conduit for relaying a number of peripheral or central signals including steroid hormones, fasting, ghrelin, leptin or photoperiod into the GnRH system (12-16). Therefore we examined the effects of morphine/naloxone injections on *KiSS1*/*GPR54* mRNA levels to investigate that if the opioids and kisspeptin pathways may interact to each other in controlling the HPG axis.

The results showed that morphine significantly down- regulated the *KiSS1* mRNA levels and naloxone blocked the inhibitory effect of morphine on *KiSS1* mRNA expression. But *GPR54* mRNA levels were not significantly influenced by morphine or naloxone injections. For the first time in reproductive axis, we investigated the effects of morphine/naloxone on *KiSS1*/*GPR54* mRNA levels and no study has previously been performed to compare this point in any species. However morphine may take part in the regulating of kisspeptin synthesis partly via other brain interneurons or peptides. It has been revealed that ghrelin system negatively influences the gonadal axis (22- 24). It has also been reported that co-administration of naloxone with ghrelin restores mean LH concentration and pulse frequency in rats (23). Moreover, ghrelin inhibits and delays the LH response to naloxone in men (24). Changes in the hypothalamic KiSS-1 system have been reported in situations of negative energy balance, which are linked to the suppressed gonadotropin secretion. Studies reported that intravenous injection of ghrelin or fasting, accompanying with increased ghrelin levels, results in a significant decrease in *KiSS1* gene mRNA level in the rat brain (16-18). Because GnRH pulse generator and kisspeptin neurons are located in the medio basal hypothalamus in which the ghrelin receptor is also expressed (25). Our studies have also shown that morphine increases hypothalamic ghrelin gene expression in male rats (data not published). Thus, central opioid system may down- regulate *KiSS1* gene expression partly via up-regulating ghrelin levels.

There is a close relationship between hypothalamus-pituitary- adrenal (HPA) and HPG axis activities. Corticotrophin- releasing factor (CRF), synthesized by hypothalamic neurons, is a potent inhibitor of the GnRH pulse generator. Central administrations of CRF decrease the GnRH concentration in hypophyseal portal system and mean plasma LH/sex steroid concentrations (26-28). While suppression of LH secretion, by CRF injection, or a variety of stressful stimuli, increasing the CRF/cortisol secretions, can be reversed by CRF antagonists (29). The previous studies have reported that injections of opioid increase CRF/ACTH release and pretreatment of the animals with opioid antagonists especially µ-type receptor antagonists abolish the inhibitory effects of CRF on GnRH/LH release, suggesting that the CRF-induced inhibition of gonadotropin secretion is mediated by opioids (27). Recently Kinsey-Jones et al. (30) showed that CRF or corticosterone injections as well as both acute and chronic stressors down-regulate *kiSS1/GPR54* mRNA levels in rat hypothalamic nuclei. So, a possible functional interaction between the opioid and CRF/corticosterone systems could be considered in regulating kisspeptin/*GPR54* signaling system. Leptin, the hormone which is mainly secreted by adipose tissue, may be involved.

Leptin is a stimulatory factor for controlling reproduction process and it improves secretion of LH hormone via projecting direct or indirect signals including kisspeptin neurons to GnRH ones (31). Studies demonstrated that kisspeptin mRNA levels are extremely lower in leptin gene knocked-out mice compared to normal ones and infusion of leptin reverse the results in these animals. They contributed to the down-regulation of HPG axis activity to declined arcuate kisspeptin levels (13). Many other studies confirmed the mediatory role of *kisspeptin/GPR54* signaling pathway for exerting leptin effects on GnRH/ LH release (31, 32). There is also an inverse relationship between plasma ß-endorphine (endogenous ligand for mu receptor) and leptin level. It has been established that ß-endorphine contains lipolytic properties and it plays an important role in decreasing body weight via declining leptin secretion (33). So, it is proposed that suppressing leptin signaling might partly be involved in the inhibitory effects of mormhine on *KiSS1* gene expression.

However for first time our results showed that down- regulation of kisspeptin pathway may have a role in the inhibitory effects of morphine on HPG axis. To better understand mechanisms of opioid-induced hypogonadism via affecting kisspeptin/*GPR54* signaling system, in further studies we could examine the effects of injection of other opiates including methadone, codeine or endogenous opioid such as ß-endorphine on hypothalamic *KiSS1/GPR54* mRNA levels. In addition, the interactions of morphine and effect of inhibitory/stimulatory factors involved in the regulation of reproduction including leptin, alpha melanocyte stimulating hormone (aMSH) or CRF should be investigated on kisspeptin/GPR54 signaling pathway and HPG axis activity.

## Conclusion

Subcutaneous injection of morphine attenuates the stimulatory effects of third cerebral ventricular injection of kisspeptin on mean plasma LH levels. Kisspeptin+naloxone exerts an additive stimulatory effect on mean plasma levels of LH compared to naloxone. Additionally, morphine significantly down-regulates the hypothalamic *KiSS1* levels and naloxone blocks the inhibitory effect of morphine on *KiSS1* mRNA expression. The GPR54 mRNA levels were not significantly influenced by morphine or naloxone injections. These results suggest that down-regulation of the kisspeptin signaling pathway might partly be involved in opioid-induced infertility.
